# Pathways, consequences, and community-proposed responses to school discontinuation in rural eastern Uganda: a qualitative study

**DOI:** 10.1080/16549716.2026.2750047

**Published:** 2026-10-21

**Authors:** Yang Jae Lee, Matthew D. Edwards, Anne C. Carrier, Charlotte Reynolds, Hernando Magallanes, Tubenawe Bruno, Marcella Nakate Ingrid, Irene Chau, Shakira Nakaweesi, Johanna Hvalić-Mwase, Gladys Namuluta, Rauben Kazungu, Sophie Waimon, Robert Rosenheck, Alexander C. Tsai, Peter Waiswa

**Affiliations:** a Department of Psychiatry and Behavioral Sciences, University of Washington, Seattle, WA, USA; b Department of Global Health, University of Washington, Seattle, WA, USA; c Empower Through Health, Jinja, Uganda; d University of North Carolina Charlotte, Charlotte, NC, USA; e Northern Michigan University, Marquette, MI, USA; f Calvin University, Grand Rapids, MI, USA; g Lewis & Clark College, Portland, OR, USA; h Cavendish University, Kampala, Uganda; i Butabika School of Psychiatric Nursing, Kampala, Uganda; j School of Public Health, Johns Hopkins University, Baltimore, MD, USA; k Department of Psychiatry, Yale University, New Haven, CT, USA; l Center for Global Health and Mongan Institute, Massachusetts General Hospital, Boston, MA, USA; m Department of Psychiatry, Harvard Medical School, Cambridge, MA, USA; n Mbarara University of Science and Technology, Mbarara, Uganda; o School of Public Health, Makerere University, Kampala, Uganda

**Keywords:** School discontinuation, school dropout, vocational support, Community-based interventions, adolescent mental health

## Abstract

**Background:**

Education is a social determinant of health, and incomplete schooling is associated with poorer sexual and reproductive health, psychosocial distress, and reduced economic opportunity. In Africa, many young people leave school before completing their educational cycle, yet qualitative evidence on the social and health consequences of discontinuation and on what affected communities view as appropriate responses remains limited.

**Objective:**

To explore perspectives on the pathways, consequences, and proposed responses to school discontinuation in rural eastern Uganda.

**Methods:**

This qualitative descriptive study drew on 59 in-depth interviews with young adults who had discontinued school (*n* = 15), family members (*n* = 14), teachers (*n* = 15), and community leaders (*n* = 15), purposively sampled with community assistance in Buyende and Kamuli districts in eastern Uganda. Data were analyzed using framework analysis.

**Results:**

Three themes emerged. First, participants described discontinuation as driven by intersecting household poverty, school-level barriers, and gendered pressures including early marriage, teenage pregnancy, and sexual exploitation. Second, discontinuation was followed by psychological distress, social stigma, economic marginalization, and heightened vulnerability, particularly for young women. Third, participants proposed complementary supports spanning counseling and mentorship, vocational training and economic empowerment, and interpersonal and community-based engagement.

**Conclusions:**

School discontinuation in rural eastern Uganda is experienced as a cumulative process with cascading social, psychological, and economic consequences rather than a single educational event. Community members identified multi-domain responses addressing emotional recovery, livelihoods, and social reintegration together. These findings highlight the value of centering community perspectives when designing integrated interventions for young people affected by school discontinuation.

## Paper Context

**Main findings:** School discontinuation in rural eastern Uganda is driven by intersecting poverty, school-level barriers, and gendered pressures, and is followed by psychological distress, stigma, economic marginalization, and heightened vulnerability for young women.

**Added knowledge:** This study extends understanding beyond the drivers of school discontinuation to document its health and social consequences and the multi-domain responses that affected communities themselves identify as necessary.

**Global health impact for policy and action:** Responses to school discontinuation should integrate counseling, livelihood support, and community-based engagement rather than treating these as separate domains, and should be embedded within existing community structures, pointing to coordinated education-health-social protection responses for affected youth.

## Background

Education is a powerful social determinant of health, with extensive evidence linking educational attainment to better health across the life course [1,2]. Yet, access to inclusive and equitable quality education remains uneven. Incomplete schooling is associated with reduced economic opportunity and adverse health and social outcomes, including higher sexual and reproductive health risk and poorer adult socioeconomic outcomes [3–5]. Thus, school discontinuation, leaving formal schooling before completing the current educational cycle and not currently being enrolled, should be understood as both an educational and public-health concern.

School discontinuation overlaps with, but is conceptually distinct from, related constructs in the literature on school attendance problems, including temporary or episodic dropout, school interruption, educational exclusion, and school refusal [6,7]. Recent work further emphasizes that school non-attendance is multidimensional and may reflect differences in emotional distress, perceived school climate, coping, social relationships, and motivation [8,9]. School discontinuation, as defined in the present study, captures sustained, frequently involuntary departure from formal schooling, where the boundary between ‘choosing’ to leave and ‘being forced’ to leave is often blurred. Thus, although school discontinuation may emerge from earlier attendance problems, it should not be treated as synonymous with school refusal or episodic absenteeism. Relatively less qualitative work from low- and middle-income settings has centered lived experience and community-prioritized responses to school discontinuation. Africa, in particular, bears a disproportionate share of the global shortfall, with some of the world’s highest rates of educational exclusion [10].

Uganda reflects these broader patterns while presenting distinct challenges. Uganda has one of the world’s youngest and most rapidly growing populations, has invested substantially in fee-free primary and lower-secondary education, yet continues to record high rates of non-completion. This juxtaposition makes the gap between expanded access and sustained participation especially visible. Major policy reforms, including Universal Primary Education, have expanded access to schooling, yet many learners still leave formal education before completing primary school or progressing through lower secondary school [11]. Studies from Uganda and other low- and middle-income settings suggest that discontinuation is shaped by intersecting structural constraints, including household poverty, indirect schooling costs despite fee-free policies, the opportunity costs of children’s labor, and gendered social pressures related to pregnancy and relationships [12–14]. Although these drivers are well documented, they have been examined primarily through quantitative analyses and policy reports.

A social-ecological perspective, which situates individual behavior within nested and interacting layers of influence spanning the individual, family, school, community, and broader societal levels, may be applied to schooling and provides a theoretical guide for this study [15,16]. This multilevel framing is also consistent with contemporary literature on school attendance problems, which increasingly conceptualizes non-attendance as arising from interactions among individual psychological factors, family and peer relationships, and characteristics of the school environment rather than from a single cause [8,9]. This perspective implies that discontinuation cannot be attributed to any single factor but instead emerges from interactions among personal circumstances, household resources, institutional practices, and prevailing social norms. Across these distinct layers of influence, many stakeholders such as family members, teachers, and community leaders may play a role in a learner’s school outcomes. Despite this, few studies that examine school discontinuation under the social-ecological model engage with external stakeholders, leaving important gaps in understanding school discontinuation at ecological levels beyond the individual.

Further, less attention has been paid to what happens after young people leave school. In Uganda, education shapes later livelihood opportunities in a labor market where many young people enter vulnerable employment and self-employment; recent policy evidence indicates that 43% of Ugandan youth are self-employed as own-account workers and 39% are not in employment, education, or training [17]. The consequences of school discontinuation are also social and gendered: Ugandan evidence identifies economic hardship and pregnancy as prominent reasons for girls’ school leaving and highlights pregnancy-related stigma as an important challenge for adolescents [18,19]. Evidence from Africa also suggests that being out of school or leaving school early may be associated with depression, anxiety, and poorer psychosocial wellbeing [20,21], yet relatively little is known about how these experiences unfold in rural settings or in the years following discontinuation. There is also limited qualitative evidence from rural Uganda on what affected communities themselves view as feasible and necessary responses, whether psychological, occupational, or relational. This perspective is important for designing interventions that address not only school retention, but also the harms that may follow school discontinuation.

Taken together, the existing literature provides useful evidence on predictors of school discontinuation, but offers less insight into how discontinuation is experienced, what social, psychological, and economic consequences follow it, and what forms of support communities consider most relevant. In particular, gaps exist in understanding differences and similarities across stakeholder groups. These gaps are especially important in rural eastern Uganda, where context-specific qualitative evidence and community-generated and accepted responses to school discontinuation remain limited. To address this gap, this study used a qualitative design in rural eastern Uganda, conducting in-depth interviews with young adults who had discontinued school and with community stakeholders, including family members, educators, and local leaders. Guided by a social-ecological framing, the study addressed three research questions: (1) What pathways lead to school discontinuation in this setting, and how do they operate across individual, household, school, and community levels? (2) What social, psychological, and economic consequences are perceived to follow discontinuation? (3) What responses do affected communities view as feasible and necessary to prevent discontinuation and to mitigate its downstream effects? By centering lived experience alongside locally generated solutions, this study provides context-specific evidence to inform more integrated responses to school discontinuation in rural Uganda.

## Methods

### Study site

This study was conducted in Buyende and Kamuli districts of eastern Uganda. According to the 2024 Uganda National Population and Housing Census, Buyende District had a population of 403,486 and Kamuli District had a population of 540,252 [22]. Fieldwork was conducted in Kagulu Sub-county in Buyende District and Kamuli Municipality in Kamuli District. Census data from the Busoga region, the greater region that encompasses Buyende and Kamuli districts, also suggest substantial educational disadvantage in the broader study setting: among persons aged 10 years and above, 21% had no formal education and 41% had only some primary education, while the literacy rate was 72% (comparable to the national average of 74% [22]).

### Study design

This study used a qualitative descriptive design [23], employing in-depth interviews (IDIs) to examine participant perspectives on school discontinuation in eastern Uganda. IDIs were used to elicit individual experiences and stakeholder-specific perspectives. A total of 59 IDIs were conducted, including 15 young adults who had discontinued school, 14 family members, 15 teachers, and 15 community leaders. This study was conducted in accordance with the principles of the Declaration of Helsinki.

### Participant eligibility and recruitment

Participants were drawn from four stakeholder groups: young adults who had discontinued school, family members of young adults who had discontinued school, teachers, and community leaders. Eligibility criteria for young adults who had discontinued school were age 18–21 years, discontinuation of school after age 12 years, and completion of at least Primary 4. For family members, teachers, and community leaders, participants were required to be at least 18 years of age. All participants were required to be from Buyende or Kamuli districts in the Busoga region of Uganda. Across all groups, individuals were excluded if they were unable or unwilling to provide informed consent. Young adults were additionally excluded if they were currently enrolled in formal schooling or fell outside the specified age range. Exact participant ages, detailed educational and socioeconomic characteristics were not collected. Other participant characteristics may be found in Table 1.

**Table 1.** Characteristics of interview participants by stakeholder group (*N* = 59).

Stakeholder group	Sex (M / F)	n
Young adults who had discontinued school	10 M/ 5F	15
Family members	3 M/11F	14
Teachers	10 M/5F	15
Community leaders	10 M/5F	15
Total	—	59

Participants were recruited through purposive sampling, with local village health team members serving as gatekeepers who identified and introduced potential participants in each of the four stakeholder categories across Buyende and Kamuli districts. Gatekeeper involvement may have influenced participant selection and favored more socially connected individuals or those more willing to discuss school discontinuation. Sampling was designed to capture the range of perspectives spanning the individual, household, school, and community levels rather than to achieve statistical representativeness. Recruitment continued within each group until the research team judged that the breadth of perspectives relevant to the study questions had been adequately captured. Reliance on gatekeeper referral may have introduced selection bias, for example, favoring individuals who were known to VHTs, more socially connected, or more willing to speak about school discontinuation. Interviews were conducted in private locations chosen at participants’ discretion.

### Data collection

Data were collected by Ugandan and American research assistants with undergraduate- or master’s-level training in social science and/or public health. Ugandan research assistants served as the primary interviewers and were fluent in English and Lusoga, the local language. American research assistants worked in pairs with Ugandan research assistants, providing observational and logistical support, including assistance with audio-recording.

Semi-structured interview guides were developed through literature review and pilot testing. Guides were organized around the study questions and addressed participants’ accounts of the pathways leading to school discontinuation, its psychosocial and economic consequences, and proposed responses, with separate but parallel versions tailored to each stakeholder group. Pilot interviews with a small number of community members led to minor revisions, including simplification and re-wording of items for cultural and linguistic clarity and reordering of questions to improve conversational flow. Pilot data were not included in the analysis. Sample interview questions are provided in Supplementary File S1. Guides were revised iteratively over the course of data collection in response to how interviews progressed and to allow follow-up of emerging topics. Before each interview, Ugandan research assistants explained the purpose of the study and obtained informed consent. Both verbal and written consent were obtained; when participants were unable to provide a written signature, fingerprints were used in its place. Because interviews could include discussion of sensitive topics, participants were reminded that they could decline any question or stop the interview at any time, and interviewers were prepared to share information about locally available support services through Mpunde Health Center if requested or needed.

IDIs lasted approximately 20 to 50 minutes. All interviews were audio-recorded, then transcribed and translated into English by the research assistants who conducted the session. To protect confidentiality, audio recordings and transcripts were labeled with study identification codes rather than participant names, and all de-identified study files were stored on password-protected, access-restricted devices accessible only to the research team. All study procedures received ethics approval prior to the start of data collection. Participants were provided with a study incentive in accordance with local norms and in-country IRB guidance. This study was approved by the Institutional Review Boards of the AIDS Support Organization in Uganda (TASO-2023–222) and Yale University (2000034605). In accordance with national guidelines, approval to conduct the study was also obtained from the Uganda National Council for Science and Technology (SS1860ES).

### Data analysis

Data were analyzed using the framework method [24] to identify recurring themes related to school discontinuation and community perspectives on its causes, consequences, and possible responses. Analysis was primarily inductive while being guided by the study aims and interview domains. Transcripts were reviewed and coded manually by pairs of Ugandan and American researchers. Disagreements between coders were handled through consensus meetings, and an audit trail of codebook revisions was maintained. In the first round of coding, researcher pairs identified recurrent concepts and preliminary themes within transcripts. A second round of coding was then conducted by a different researcher pair to provide an additional interpretive perspective on the data.

After approximately two to three interviews per participant category had been completed and coded, the research team developed a preliminary codebook. The codebook organized major themes, subthemes, and individual codes and served as the analytic framework for subsequent coding. As additional transcripts were reviewed, the codebook was revised iteratively through regular team discussions to incorporate newly emerging ideas and refine thematic definitions. After data collection was complete, the codebook was further condensed and refined to reflect the study’s final thematic structure. Data collection proceeded until thematic saturation was reached in each subgroup. Thematic saturation was defined as the point at which recurring themes were observed across interviews and additional interviews did not yield substantively new themes. The resulting coding framework, comprising the major themes, subthemes, and representative codes, is presented in Table 2.

**Table 2.** Coding framework: themes, subthemes, and representative codes.

Theme	Sub-theme	Representative codes
1. Pathways to school discontinuation	Economic and household drivers	Household poverty; inability to pay fees and indirect costs; child labor and income-generating work; household caregiving responsibilities
	School- and community-level drivers	Unsupportive school environment; focus on fee payment; punitive discipline and public shaming; curriculum inconsistency; limited community or extended-family support
	Gendered drivers	Teenage pregnancy; early marriage norms; menstrual hygiene barriers; sexual exploitation by older men
2. Consequences of school discontinuation	Psychological distress and social stigma	Sadness, regret, and shame; social withdrawal; being judged, avoided, or treated as morally suspect; substance use and petty crime
	Economic marginalization and barriers to reintegration	Idleness; precarious or exploitative informal work; perpetuation of poverty; difficulty re-enrolling
	Gendered vulnerability after discontinuation	Sexual coercion; unstable relationships; abandonment; single motherhood; difficulty returning after pregnancy
3. Community-proposed responses	Counseling and mentorship	Need for counseling and guidance; career mentorship; role modeling; community sensitization on the value of education
	Vocational and economic support	Vocational and skills training; re-enrollment scholarships; youth savings groups; startup capital for small businesses
	Interpersonal and community-based support	Family and caregiver encouragement; school clubs and sports; locally embedded support structures; reducing stigma between in- and out-of-school youth

Several strategies were used to enhance the trustworthiness of the findings, drawing on established criteria for qualitative rigor [25]. Credibility was supported through investigator triangulation, with transcripts coded by two researcher pairs and discrepancies resolved through consensus discussion. Data‑source triangulation across the four stakeholder groups allowed convergent and divergent perspectives to be examined. Dependability was addressed by maintaining an audit trail documenting codebook development and successive revisions. Confirmability was supported by grounding interpretations in participant accounts and by team-based review of coding decisions, which reduced the influence of any single coder.

### Research team and reflexivity

The research team included both Ugandan and American researchers participating in the Global Health Experiential Fellowship (GHEF), a structured global health training program [26]. Ugandan research assistants led interviews in Lusoga or English and contributed local linguistic and cultural knowledge that supported contextual interpretation. American research assistants played an observational and supportive role during data collection and participated in coding and analytic discussions. The team recognized that differences in nationality, language, educational background, and institutional affiliation could influence participant disclosure and analytic interpretation, such as varied opinions about codes. Using Ugandan interviewers as primary facilitators was intended to reduce some of these barriers, while team-based coding and regular discussion were used to reflect on potential power imbalances throughout analysis.

## Results

A conceptual framework of school discontinuation and its consequences situated within a social ecological model was guided by findings from IDIs with multi-level stakeholders (Figure 1). Representative quotes and codes are presented in Table 3.

**Figure 1.**
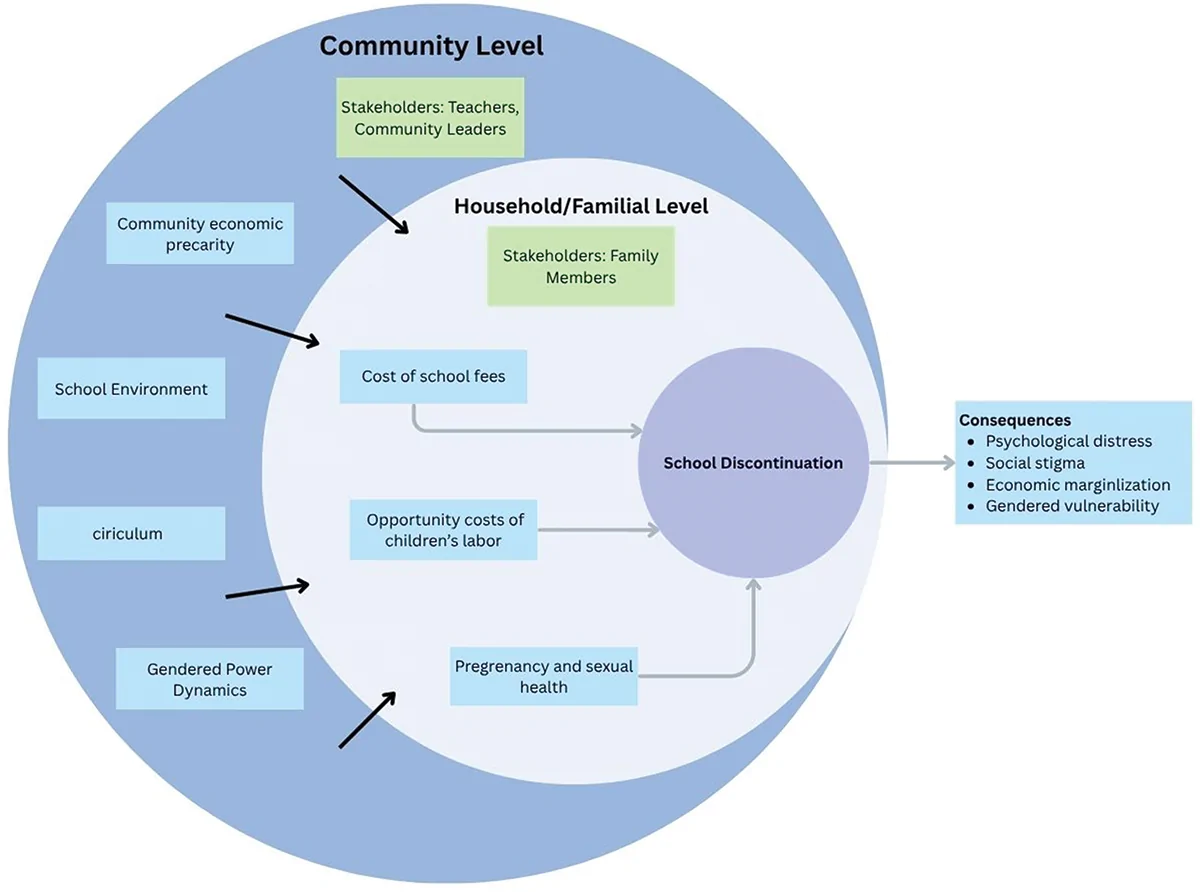
Conceptual framework for school discontinuation in rural eastern Uganda across social-ecological levels.

**Table 3.** Illustrative coding examples linking participant quotations to codes, subthemes, and themes.

Representative quotation	Code	Sub-theme	Theme
‘Leaving school was never my personal choice. It was just because my mother never had money … if she was financially stable, I would be in school even now.’ – IDI, man who discontinued school	Poverty; inability to pay fees	Economic and household drivers	Pathways
‘For the case of girls, they discontinue because of early teenage pregnancy; when she conceives, she drops out … for boys, it’s not too common.’ – IDI, man, teacher	Teenage pregnancy	Gendered drivers	Pathways
‘He used to be in the house and keep quiet … when the friends were going to school in the morning, he would sleep until everyone going to school disappeared.’ – IDI, woman, family member	Withdrawal; shame	Psychological distress and social stigma	Consequences
‘Those who are not at school are the ones that are always there without anything to do – it makes them remain idle.’ – IDI, man, family member	Idleness; lack of work	Economic marginalization	Consequences
‘This male promised to take me back to school after delivering the baby but then he left … Now I am a single mother like my mother.’ – IDI, woman who discontinued school	Abandonment; single motherhood	Gendered vulnerability	Consequences
‘There is no counseling or guidance for these children. No one helps them to understand that they still have a future.’ – IDI, man, teacher	Need for counseling and guidance	Counseling and mentorship	Responses
‘I think building vocational schools would be helpful in the community. These schools can train children in life skills … such as driving, hairdressing, tailoring … to start businesses and help their future.’ – IDI, man who discontinued school	Vocational and skills training	Vocational and economic support	Responses
‘Games like football, volleyball, or ludo can bring youths together – both those in school and those who discontinued – and help prevent stigma and isolation.’ – IDI, man who discontinued school	Clubs and extracurriculars	Interpersonal and community-based support	Responses

### Theme 1: pathways to school discontinuation

Across stakeholder groups, participants described school discontinuation as shaped by intersecting economic, household, school, community, and gendered influences. Rather than attributing discontinuation to a single cause, participants described it as emerging through cumulative pressures that made continued schooling difficult to sustain.

#### Sub-theme 1: economic and household constraints

Household poverty was a commonly cited driver of school discontinuation. Participants described a recurring pattern in which inability to pay school fees led to repeated dismissal from school and, eventually, permanent withdrawal. As one young man explained,

Leaving school was never my personal choice. It was just because my mother never had money … if she was financially stable, I would be in school even now. - IDI, man who discontinued school.

Financial strain also pushed children into income-generating labor that competed with schooling. Teachers described children being used in household gardens or hired out for wages, contributing to fatigue, absenteeism, and disengagement. As one teacher stated,

Child labor is also another problem … some parents using children as laborers at their family farms, gardens, and also other people so that they get money. - IDI, man, teacher.

Participants also described family disruption as undermining school continuity. Parental separation, caregiver burden, and domestic conflict were said to reduce households’ capacity to keep children in school. One parent described reaching a point of exhaustion:

I reached a time and I felt overburdened from taking care of all the children myself. - IDI, woman, family member.

Beyond financial hardship, participants described household expectations regarding the value and duration of schooling. Some family members viewed formal education as worthwhile only up to a certain point, after which vocational work was seen as a more realistic option. As one father explained:

It depends on the capacity of the parents … at least the child should be educated up to S4 and then … be taken to vocational training. - IDI, man, family member.

Family members also described the absence of broader community or extended-family support for school fees, leaving individual households to manage these costs alone:

You see here in the community, everyone takes care of their children, none of them can think of supporting your child … not even your family member. - IDI, woman, family member.

#### Sub-theme 2: school and community environments

Participants described school and surrounding community environments as additional contributors to discontinuation. Teachers and community leaders reported overcrowded classrooms, insufficient teaching staff, lack of textbooks and furniture, and inadequate provision of meals. Hunger during the school day was described as one factor that could reduce children’s ability to remain engaged. As one community leader noted:

I also heard that some children don’t eat and starve for the full day so I think even this can lead to school discontinuation. - IDI, man, community leader.

Curriculum inconsistency was described as another barrier, particularly for students transferring between schools. One teacher explained:

If a student changes from one school to another, they will find that the school teaches a different syllabus from the one they left and they will fail to catch up hence discontinue school. - IDI, man, teacher.

Young adults who had discontinued school often described schools as unresponsive to their circumstances and focused primarily on fee payment. One female participant stated:

I didn’t receive any help from the school, the school didn’t really care. All they needed was their money. - IDI, woman who discontinued school.

Teachers also acknowledged that punitive disciplinary practices, including public shaming of students who had not paid fees, could intensify disengagement. As one teacher explained:

They bring that boy or girl to the assembly yet they are going to be ashamed … enough can make the boy or girl discontinue. - IDI, woman, teacher.

#### Sub-theme 3: gendered drivers

Participants across stakeholder groups consistently described pregnancy, marriage norms, menstrual‑hygiene challenges, and sexual exploitation as drivers of discontinuation that disproportionately affected girls. Early marriage was described as a prominent pathway out of school, with participants attributing it to both parental pressure and wider community norms that valued marriage over continued education for girls. As one mother explained:

So some of them encourage young girls to get married at an early age. - IDI, woman, family member.

Teenage pregnancy was also described as a major cause of discontinuation. Participants noted that pregnancy often resulted not only in withdrawal from school but also in stigma that made return difficult. One teacher stated:

For the case of girls, they discontinue because of early teenage pregnancy; when she conceives, she drops out … for boys, it’s not too common. - IDI, man, teacher.

Lack of menstrual hygiene products was described as another barrier to girls’ school attendance and engagement. Teachers reported that repeated difficulty managing menstruation at school contributed to absenteeism and gradual disengagement. As one teacher explained:

When they get to the time of their periods, they usually experience difficulties because they don’t have materials to use … they start to have a negative attitude towards school and end up dropping out. - IDI, man, teacher.

Participants also described sexual exploitation by older men as a mechanism through which economic vulnerability translated into school leaving for girls. Material support such as food, money, or sanitary products was said to make girls more vulnerable to coercive or unequal relationships. One teacher explained:

These girls are more likely to be deceived by these outside men … one of them can admire a girl and buy for them their underwear … let me just go and get married, that is it. - IDI, woman, teacher.

Across these accounts, participants described school discontinuation as resulting not from a single event but from the cumulative interaction of household poverty, gendered norms, and school and community environments that did not accommodate these vulnerabilities.

## Theme 2: consequences of school discontinuation

Participants described school discontinuation as followed by psychological distress, social exclusion, and economic hardship. They also described gendered risks after leaving school, particularly for young women.

### Sub-theme 1: psychological distress and social stigma

Family members and young adults described marked emotional changes following discontinuation, often linked to seeing former peers continue in school. One mother recounted her son’s withdrawal:

He used to be in the house and keep quiet … when the friends were going to school in the morning, he would sleep until everyone going to school disappeared from home. I really felt like he still wanted to go to school, but I had nothing to do about it. - IDI, woman, family member.

Young adults who had discontinued school described feelings of sadness, regret, shame, and frustration. One young man stated:

I experienced sadness, regret, stress and felt so bad … I never even wanted to look at my mother … I reached a time of regret of being born in a poor family. I never had peace. – IDI, man who discontinued school.

Participants also described stigma at both interpersonal and community levels. Young adults reported avoiding former classmates out of shame, while teachers and family members described broader social exclusion of out-of-school youth. One teacher explained:

You may find those who have stopped schooling … their parents or community members do not want them to associate with their children who are still in school because they consider them bad people. - IDI, woman, teacher.

Substance use and petty crime were perceived by some participants to be additional consequences of idleness, peer pressure, and emotional distress among out-of-school youth. One participant reported:

They never got a living after leaving school; they joined groups and they only take drugs. - IDI, man who discontinued school.

Community leaders described these behaviors as further intensifying stigma and mistrust. As one community leader explained:

Sometimes community members try to give these boys some work … but they steal their stuff and run away. These boys use drugs - these drugs cause loneliness, too much worrying, and isolation. - IDI, woman, community leader.

#### Sub-theme 2: economic marginalization and barriers to reintegration

Participants described school discontinuation as often followed by prolonged idleness or entry into precarious informal work. Family members emphasized that many young people who left school lacked both skills and viable livelihood options. As one father stated:

Those who are not at school are the ones that are always there without anything to do - it makes them remain idle. - IDI, man, family member.

When young people did obtain work, it was often described as exploitative and poorly paid. One mother noted:

He was given a lot of work to do but not even getting paid or little money. - IDI, woman, family member.

Participants who had accessed vocational training or informal apprenticeships, such as tailoring, hairdressing, or car repair, described more favorable outcomes. Stakeholders presented these opportunities as important alternatives for youth who had already left school. As one teacher observed:

When a child who dropped out of school is skilled, they don’t struggle to survive … theycan set up small businesses and will not live blaming themselves for leaving school. – IDI, man, teacher.

At the same time, access to vocational training was itself described as constrained by cost and distance. One community leader explained:

Most of the vocational training here is expensive, and sometimes people might not be able to afford transport to go to those schools. - IDI, woman, community leader.

Some young people attempted to return to formal schooling, but participants described re-enrollment as socially difficult, particularly for older students and for those who had children. A teacher explained:

Some are received well by peers and some are not … those who have children are called mothers and fathers of the school - it sometimes leads to depression and anxiety. - IDI, man, teacher.

#### Sub-theme 3: gendered vulnerability after discontinuation

Participants described distinct risks for girls after leaving school. Some reported sexual coercion or exploitation in the period following discontinuation. As one male participant who had discontinued school stated:

For girls, they treat them badly because … boys who admire them … decide to rape her. - IDI, man who discontinued school.

Other female participants described entering relationships in search of economic stability or promised educational support, only to be abandoned. One young woman recounted:

This male promised to take me back to school after delivering the baby but then he left … Now I am a single mother like my mother. - IDI, woman who discontinued school.

Across these accounts, participants described discontinuation as increasing vulnerability not only to economic hardship but also to unstable relationships, stigma, and gendered forms of harm.

## Theme 3: community-proposed supports

When asked what forms of support would be most useful, participants identified three broad and complementary domains: psychological and counseling support, vocational training and economic empowerment, and interpersonal and community-based support.

### Sub-theme 1: psychological and counseling support

Participants across stakeholder groups described the emotional consequences of discontinuation as an important barrier to moving forward after discontinuation and viewed counseling as a foundational intervention. Participants described young people who had lost a sense of purpose after leaving school. As one teacher explained:

There is no counseling or guidance for these children. No one helps them to understand that they still have a future. - IDI, man, teacher.

Young adults who had discontinued school also called for counseling to be integrated into school programming, emphasizing the need for mentorship and career guidance before disengagement occurred. One participant stated:

I would incorporate counseling programs in school … most students lack guidance. They don’t even know what career path to take. But if they are mentored and counseled in what to do, they can concentrate on their education and fulfill their dreams. - IDI, woman who discontinued school.

Teachers and community leaders also described the value of role modeling and community sensitization. They emphasized sharing examples of students from poor backgrounds who had succeeded through education and encouraging parents to see education as worthwhile. As one teacher recounted,

She was from a very humble family, but with encouragement she got a bursary and now studies medicine for free. - IDI, woman, teacher.

Another teacher highlighted the value of sensitization:

Sensitizing the value of education for parents and learners … we tell the values of education to both, and that can be good. - IDI, woman, teacher.

Several participants, therefore, emphasized that counseling and guidance should be made more accessible through existing schools and community structures.

### Sub-theme 2: vocational training and economic empowerment

Vocational training was among the most frequently proposed interventions across stakeholder groups. Participants described it as important both for preventing disengagement among at-risk youth and for helping those who had already left school achieve greater self-reliance. Family members emphasized both demand for vocational programs and the financial barriers to accessing them. As one mother stated:

I think vocational programs should be put in place, especially for parents who can’t afford fees. Most of these children have an interest … but they can’t really do it because of money. - IDI, woman, family member.

Young adults who had discontinued school similarly described vocational schools as relevant to local needs. One participant explained:

I think building vocational schools would be helpful in the community. These schools can train children in life skills … such as driving, hairdressing, tailoring … to start businesses and help their future. - IDI, man who discontinued school.

Participants also proposed re-enrollment scholarships for academically strong youth, youth savings groups, and startup capital for small businesses as broader forms of economic support. One father suggested:

There could also be initiatives such as giving people some startup capital … so that these children can use small businesses as a means of survival. - IDI, man, family member.

Although government and NGO support was occasionally described, participants characterized existing assistance as inconsistent and short-lived. One mother explained:

My first child was being supported by an NGO … they used to pay school fees and send books, but only for two years. Then it stopped. - IDI, woman, family member.

These experiences contributed to participants’ preference for more sustainable and locally embedded forms of economic support.

### Sub-theme 3: interpersonal and community-based support

Participants identified family members, teachers, mentors, and peers as important sources of support for both school retention and post-discontinuation recovery. Caregiver advocacy was described as especially important, even when families had limited material resources. One mother explained:

I supported them by paying fees, and I used to plead with the teachers to let him study until I could pay. They used to listen to me, but later I couldn’t plead anymore. - IDI, woman, family member.

Teachers described extending their roles beyond classroom instruction to include encouragement, follow-up, and mentorship. As one teacher stated:

I encourage them to study hard and tell them that all the good things they want to get are in the future … if they think of discontinuing school, I tell them they won’t fulfill their dreams. – IDI, man, teacher.

Participants also proposed community mentorship programs that would provide young people with relatable examples of educational success. One teacher explained:

We advise them and give them examples of people who have studied and are now well, so they can admire to be like them. - IDI, woman, teacher.

Extracurricular activities, including school clubs and sports, were described as additional ways to strengthen connections and reduce stigma between in-school and out-of-school youth. One young man explained:

Games like football, volleyball, or ludo can bring youths together - both those in school and those who discontinued - and help prevent stigma and isolation. - IDI, man who discontinued school.

Teachers also described clubs as informal spaces for counseling and follow-up, including for girls at risk of early marriage. As one teacher stated:

The club does activities that demonstrate the importance of staying in school … if a child is being disturbed in the community or told to get married, the club patrons can counsel and follow up at their homes. - IDI, man, teacher.

Across these accounts, participants described proposed supports as complementary rather than interchangeable. Counseling, livelihood support, and relational support were each seen as necessary, but insufficient on their own.

## Convergence and divergence across stakeholder groups

Although the three themes were shared across the sample, the four stakeholder groups emphasized them differently. There was broad convergence on the primary driver of school discontinuation among all stakeholder groups that household poverty and the cost of schooling as the central reasons young people left school. The groups also converged in their preferred response, with vocational training and economic support proposed across all four perspectives as a realistic way to support young people who had discontinued school. Divergence was most apparent in where each group located the problem. Young adults emphasized schools’ indifference to their circumstances and the internal experience of shame and distress that followed leaving school. Teachers tended to foreground structural and behavioral factors within and around schooling, including the competing demands of household labor, school discipline, the relevance of the curriculum, and the management of menstruation for girls. Family members framed discontinuation primarily in terms of household economic burden and a pragmatic calculation about the value of continued education relative to its cost. Community leaders most often emphasized downstream community-level consequences, including idleness, substance use, and crime, as well as the stigma attached to young people who had left school.

## Discussion

This qualitative study examined how young adults who discontinued schooling in rural eastern Uganda, together with family members, teachers, and community leaders, understood the causes and consequences of school discontinuation and the supports communities considered most relevant. Three findings emerged. First, participants described discontinuation as resulting from the cumulative interaction of household poverty, school and community barriers, and gendered norms, particularly those related to pregnancy, early marriage, menstrual hygiene, and sexual exploitation. Second, they linked discontinuation to psychological distress, social stigma, economic marginalization, and gendered vulnerability. Third, they proposed a multidomain response combining counseling, vocational and economic assistance, and interpersonal and community-based support. Together, these findings position school discontinuation not as a single educational event, but as a social-ecological process with cascading psychosocial and economic consequences across individual, household, school, and community levels.

The pathways described by participants are broadly consistent with evidence from Uganda. National and Uganda-specific studies have identified household poverty, indirect schooling costs, child labor, and place-specific disadvantage as important drivers of school discontinuation [11,12,14]. The gendered patterns in our data, particularly pregnancy-related withdrawal, stigma surrounding school re-entry, and vulnerability shaped by material deprivation, also align with Ugandan research on pregnancy-related stigma and young people’s transitions into risky or economically constrained relationships [13,18]. Young adults and teachers tended to foreground gendered and social determinants of school discontinuation, suggesting that those with lived experience of how social norms shape everyday school trajectories may better inform interventions aimed at upstream social factors. Recent qualitative research similarly underscores the role of community norms and local social environments in shaping schooling trajectories [27]. Rather than identifying entirely new drivers, the findings deepen understanding of how these pressures are experienced as intersecting within a social-ecological framework and mutually reinforcing in one rural setting. The more novel contribution of this study lies in its account of what follows discontinuation.

Participants described leaving school as the beginning of a cascade, or proliferation of stressors [28], including emotional distress, shame, community exclusion, loss of peer networks, constrained livelihood options, and heightened vulnerability. These findings demonstrate that, like factors influencing discontinuation, the psychological consequences of discontinuation interact at different levels of the social ecological framework, from individual stressors to community-level social stigma. Research from Tanzania has documented elevated depression, anxiety, and psychosocial distress among out-of-school adolescent girls and early school leavers [20]. Our findings extend this literature by illustrating how such consequences are experienced and interpreted in everyday life in rural Uganda, particularly among different stakeholder groups. Evidence from the same district indicates that community members recognize both economic and psychosocial causes of mental distress and favor community-level support where formal services are limited [29]. Participants’ accounts of stigma further illustrate the social consequences of discontinuation, with out-of-school youth described by community leaders as avoided, judged, or treated as morally suspect, while some young participants reinforced this, describing how they withdrew from peers because of embarrassment. These experiences are consistent with prior evidence linking early school leaving to diminished psychosocial well-being [20]. Gendered vulnerabilities were especially pronounced. Participants connected school discontinuation with sexual coercion, unstable relationships, abandonment, and difficulty returning to school after pregnancy, consistent with research on pregnancy-related stigma in rural Uganda [18] and evidence from Southern Africa associating school dropout with higher HIV and HSV-2 incidence among young women [5].

A second contribution is the community-generated framework of support that emerged from participants’ accounts. Participants across stakeholder groups viewed psychosocial, relational, educational, and livelihood needs as interdependent and emphasized responses embedded within existing community structures. They described counseling, livelihood assistance, and relational and community-based support as complementary rather than interchangeable. This multisectoral emphasis aligns with evidence that interventions addressing school completion and adolescent mental health often span multiple domains [30,31], as well as with regional approaches to multisectoral HIV responses [32–35]. Emotional recovery, economic viability, and social support were understood as mutually reinforcing conditions for re-engagement, targeting multiple domains across the social-ecological model. Participants specifically identified teachers, mentors, clubs, caregivers, and local training opportunities as potential platforms for support. This preference is consistent with community-based Ugandan programs combining psychosocial, educational, and livelihood assistance for vulnerable young women [36] and with calls for adolescent mental health services in sub-Saharan Africa that are accessible and less dependent on scarce specialists [37].

This study has several strengths. Interviews with young adults who had discontinued school, caregivers, teachers, and community leaders provided multiple perspectives on the same topic. The study also examined pathways to discontinuation, post-discontinuation consequences, and responses proposed by the community, yielding an integrated account of a phenomenon often studied more narrowly. Several limitations should be considered. First, the cross-sectional design relied on retrospective accounts and may be affected by recall and social desirability biases. Recruitment through community health workers may also have favored youth who were more visible, socially connected, or known to community structures, thereby underrepresenting those who were highly marginalized or socially isolated from local systems. Second, the study did not collect exact ages or systematic demographic information beyond eligibility screening, including time since discontinuation and later circumstances such as partnership, household formation, or parenthood. This limits assessment of within-group variation and of outcome differences by age or time since leaving school. Third, the study was conducted in two sites in the Busoga region; transferability to other Ugandan settings or countries should not be assumed. Translation of interviews may have reduced linguistic nuance, and preliminary findings were not returned to participants for member checking, limiting direct confirmation of interpretive accuracy. Finally, although Ugandan researchers led data collection, American researchers participated in observation and analysis, and cross-national power dynamics may have influenced disclosure and interpretation.

Viewed through a social-ecological lens, these community-informed findings inform implications and cross-sector recommendations for addressing school discontinuation. Such recommendations, however, do not establish program efficacy. First, educational policy should address the household economic pressures and gendered risks surrounding schooling, not only formal tuition costs. Schools may need more responsive and supportive environments, including menstrual hygiene resources and clear re-entry pathways for students who leave because of pregnancy or other disruptions. The broader SAP intervention literature similarly suggests that attendance problems often require responses extending beyond a single individual-level intervention [38]. However, because much of this evidence concerns school refusal, absenteeism, or truancy among currently enrolled students, its applicability to young people who have already discontinued schooling in resource-constrained rural settings remains uncertain. This may be addressed better through community organizations, which could strengthen locally embedded mentorship, clubs, and vocational opportunities as platforms for support. Mental health services should be accessible, community‑based and integrated with educational and livelihood assistance rather than delivered in isolation.

Overall, responses to school discontinuation in rural eastern Uganda may be most effective when they address economic hardship, school and community environments, and gendered barriers together. The prominence of indirect schooling costs, despite fee-free policy frameworks, reinforces longstanding evidence that household-level barriers extend well beyond tuition [14].

Future research should use longitudinal designs to follow young people after school discontinuation and mixed-methods or intervention studies to test whether community-embedded, multicomponent approaches improve mental health, livelihood, and educational outcomes. Given the prominence of gendered pathways and consequences, studies focused on young women’s post-discontinuation experiences, including re-entry after pregnancy, are especially needed. Research should also examine how trajectories change over time, compare integrated and single-domain interventions, and assess which patterns are setting-specific versus broadly shared across regions and countries.

## Conclusion

This study provides qualitative evidence from rural eastern Uganda on how school discontinuation occurs, what follows it, and what communities view as necessary responses through a multi-stakeholder lens. Participants described discontinuation as emerging from intersecting economic, institutional, and gendered pressures and as followed by distress, stigma, economic marginalization, and gendered vulnerability. They also proposed an integrated support framework spanning counseling, vocational and economic support, and community-based relational support. These findings underscore the value of centering a range of stakeholder and community perspectives when addressing school discontinuation as a social and public-health concern. They also suggest that governments and implementing partners should move beyond narrow retention-focused strategies and consider integrated responses that address the psychosocial, educational, and economic consequences of school discontinuation together.

## Supplementary Material

SRQR Checklist 917.docx

Supplementary File 1 .docx

## Data Availability

Data are available upon reasonable request to the corresponding author.
