# Supplementary material for: Pathways, consequences, and community-proposed responses to school discontinuation in rural eastern Uganda: a qualitative study
Source: Glob Health Action. 2026 Oct 21;19(1):2750047. doi: 10.1080/16549716.2026.2750047 (PMC13689791; doi:10.1080/16549716.2026.2750047)
Supplement: Supplementary file 1 [file ZGHA_A_2750047_SM4639.docx]

**Semi-Structured In-depth Interview Guide for Community Leaders**

**Topic:** Retrospective interviews to investigate how life looks like for children after leaving school, community attitudes, stigma experiences and psychological impacts. Specifically, parents or caregivers of youth who left school early are asked to provide deeper insights into the topic.

***Omulamwa:*** *Ebibuuzo eby’okunonereza ku nsonga edha okuvha kwiisomero amangu, endowooza y’ekyaalo, okweinhamiza okubaawo ne engeri yekikyusa embera y’obuntu. Okusinga, abazaire oba abalabirila abavhubuka abaava kwiisomero amangu babuziibwa okugha ebisingaawo kumulamwa guno*.

**Introduction of the Researcher**

Hello. I am…..from Empowerment to Heal Uganda, based at Mpunde Health Center. Our team is conducting a study in Buyende to understand the reasons for leaving school early and the stigma attached to it. The interview will take approximately 45 minutes. Please feel free to respond to the questions openly; there are no right or wrong answers. All your responses will be recorded. I will record the interview to ensure that I capture all details accurately, and it will be transcribed later.

*Mbalamwiisa. Ninze...............................okhuva mu Empowerment to Heal Uganda, okuvha kw’ilwaliro lye Mpunde. Ekitongole kyaiffe kili kola omusomo mu Buyende okutegera ensonga lwaki abaanha bavha kw’isomero amanghu n’obwewalikirivu obwekosa kw’ekyo.*

*Ebibuuzo bida kumala akabhanga akagere ka dakkika 45. Wulira eidhembe okwilamu ebibuuzo mulwangala, tuzila kyakwilamu kituffu oba kiffu. Eby’okwilamu byaimwe byona byona bidhakugemebwa. Ndigya gema amalobozi okusobola okugeman buli kagema ku bibuuzo byaiffe obulunji, oluvhainhuma mbiize mubuwandhiike.*

**Location**

District

*Disitulikiti*

County

*Isaaza*

Subounty

*Igombolola*

Parish

*Muluka*

Village

*Kyaalo*

**Background information**

Name

*Elinha*

Sex

*Ekikula*

Occupation

*Omulimo*

Marital status

*Embera y’obufumbo*

Education Level

*Okusoma*

Religion

*Eidhini*

Phone number

*Eisimu*

Section 1: Community Views on School Discontinuation

1. From your perspective, what are the main reasons children in your community leave school early?

*Okuva mundowooza yo, nsonga kyi enkulu lwaki abaanha mukitundhu kyo bava kwiisomero amangu?*

2. Are there specific groups (girls, orphans, children with disabilities) more likely to discontinue school? Why?

Probe: Are boys and girls treated differently in the community after discontinuing school?

*Eliiyo ebibonondho ebitegerekesse (abawala, abalenzi, bamulekwa, abanha abaliku obulema kumubiri) ababonekeire okuvha kwiisomero ? Lwaki*?

Buuza: Abaana abawala n’abalenzi babisibwa ky’ekimu nga balekeire okusoma?

3. How does the community typically respond when a child discontinues school?
   a. Probe: judgement, support, indifference

*Ekyaalo ky’ebisa kitya nga omwana aviile kwiisomero?*

*a) Buuza: Babasalira emisango, bayambibwa, tibibagemaku*

4. How do families react to children after they leave school?

Probe: Do they give any support?

*Ab’engandha bayamba bebisa batya ngha omwanha aviile kwiisomero?*

*Buuza: Balina obuyambi bwe babawa?*

Section 2: Observed Outcomes and Needs

5. What usually happens to children in this community after they leave school early?
   a. Probe: Do they find work, get married early, become isolated, migrate?

*Kiki ekitera otuuka kubaanha mukitundhu muno nga baviile kwiisomero amangu?*

*a) Buuza: bafuna emirimo, bafumbirwa, babasosola, basenghuka?*

6. Do you think they face challenges in finding jobs or participating in the economy?

*Olowooza bagana obuzibu mukufuna emirimo oba okwetaba muby’amaguzi?*

7. Do you think school discontinuation affects the mental health or self-esteem of these youth? How does it affect them?
   a. Probe: any observed depression, substance use, or withdrawal.

*Olowooza okuva kwiisomero kikosa obulamu bw’akawanga oba embeera dh’abavubuka bano? Bubakosa butya?*

*a) Buuza: okwewalikirila kwewabonaku, okozesa ebilagalalagala, oba okwewala*

Section 3: Existing and Needed Support Systems

8. Are there any programs or systems currently supporting youth who have discontinued school?
   a. Probe: NGOs, church groups, savings groups, counseling

*Eliyo entekateka oba emiwatwa edhiri kuyamba abavhubuka abaavha kumasomero?*

*a) Buuza: ebitongole, bubondho bwa kanisa, bubondho bwa tereka sente, kw’ogera nabo*

9. What do you think are the biggest gaps in support for these individuals?

Probe: Government initiatives, NGOs, Vocational institutions.

*Olowooza abangha amanene galiwa mukuyamba abantu oti nibanho?*

*Buuza: ntekateka da government, bitongole, matendekero ga mirimo gya mumutwe.*

10. How does the community view vocational training programs or alternative education options?
    a. Probe: Are they seen as valuable? Accessible?

*Ekyaalo kibona kitya entekateka gy’emisomo gy’omumutwe oba okusoma okw’engeri eyindhi?*

*a) Buuza: babibona ng’ebyomugaso? ebitukibwaku?*

11. How open do you think families would be to youth joining local vocational and mental health programs?

*Olowooza ab’engandha bandibaire baikiriza kyagawa okwikiriza abavhubuka okwegeitta mukusoma emirimo gy’omumutwe n’obulamu bw’akawanga?*

Section 4: Recommendations and Reflections

12. What kinds of programs or services do you believe would be most helpful to youth who leave school early?
    a. Probe: job skills, mental health counseling, vocational training

*Ntekateka kika kyi oba emirimo dholowooza ngha n’egyisinga kuyamba abavhubuka abavha kwiisomero amangu?*

*a) Buuza: kutendekebwa bukodyo bwa murimo, kwogeraku bulamu bw’akawanga, kutendekebwa mirimo gya mumutwe*

13. What role do you think the community (or leaders like you) could play in making these programs successful?

*Mulimo kyi gw’olowoza ekyaalo (oba abakulembeze nga iwe) gye basobola okola okuyamba entekateka dhino okubitamu?*

14. If you could change one thing to reduce school discontinuation or improve life outcomes afterward, what would it be?

*Singa osobola okukyusayo ekintu ilala okukendeza okuvha mwiisomero oba okuyamba embera y’obulamu mumaiso eyo, kyandhibeire ki?*

**Semi-structured In-depth Interview Guide for Parents or Caregivers of Youth who Discontinued Education**

**Topic:** Retrospective interviews to investigate the reasons for leaving school, community attitudes, stigma experiences and psychological impacts. Specifically, parents or caregivers of youth who left school early are asked to provide deeper insights into the topic.

***Omulamwa:*** *Ebibuuzo eby’okunonereza ku nsonga edha okuvha kwiisomero/okulekera okusoma amangu, endowooza y’ekyaalo, okweinhamiza okubaawo ne engeri yekikyusa embera y’obuntu. Okusinga, abazaire oba abalabirila abavhubuka abaava kwiisomero amangu babuziibwa okugha ebisingaawo kumulamwa guno.*

**Introduction of the Researcher**

Hello. I am…..from Empowerment to Heal Uganda, based at Mpunde Health Center. Our team is conducting a study in Buyende to understand the reasons for leaving school early and the stigma attached to it. The interview will take approximately 45 minutes. Please feel free to respond to the questions openly; there are no right or wrong answers. All your responses will be recorded. I will record the interview to ensure that I capture all details accurately, and it will be transcribed later.

*Mbalamwiisa. Ninze...............................okhuva mu Empowerment to Heal Uganda, okuvha kw’ilwaliro lye Mpunde. Ekitongole kyaiffe kili kola omusomo mu Buyende okutegera ensonga lwaki abaanha bavha kw’isomero amanghu n’obwewalikirivu obwekosa kw’ekyo.*

*Ebibuuzo bida kumala akabhanga akagere ka dakkika 45. Wulira eidhembe okwilamu ebibuuzo mulwangala, tuzila kyakwilamu kituffu oba kiffu. Eby’okwilamu byaimwe byona byona bidhakugemebwa. Ndigya gema amalobozi okusobola okugeman buli kagema ku bibuuzo byaiffe obulunji, oluvhainhuma mbiize mubuwandhiike.*

District

*Disitulikiti*

County

*Isaaza*

Subounty

*Igombolola*

Parish

*Muluka*

Village

*Kyaalo*

**Background information**

Name

*Elinha*

Sex

*Ekikula*

Occupation

*Omulimo*

Marital status

*Embera y’obufumbo*

Education Level

*Okusoma*

Religion

*Eidhini*

Phone number

*Eisimu*

**Section 1: Background & School Discontinuation**

1. How many children do you have, and how many have left school early?

*Olina abaanha bameka, era bameka abavha kwiisomero amangu?*

2. Can you tell me about your child/children who left school—what level were they in when they stopped? Were they a boy or a girl?

*Osobola okunkobera ku mwana oba abaanha abaava kwiisomero—yali kumutendera ki weyalekerela? Yali mulenzi, oba muwala?*

3. Was it their personal choice to leave school, or outside factors influenced that decision?

*Kwali kusalawo kwaibwe okuva kwiisomero,oba muntu wundi neyaletera okusalawo kwaibwe?*

4. What were the main reasons they left school? ( Specify reason for each child)
   a. Probe: financial hardship, pregnancy, caregiving, illness, academic struggles

*Nsonga ki enkulu lwaki yava kwiisomero? (Yogeraku ku nsonga edha buli mwana)*

*a) Buuza: bukalubirivu bwa sente, yalindha, kulabirila bandhi, bulwaire, ntalo dha kusoma*

5. Did family try to support this child/children to stay in school? If so, how?

Probe: Was there anything hindering you as a person to support your child/children to stay in school?

*Wagezaaku okumuyamba okusigala mwiisomero? Oba wamuyamba, otya?*

*Buuza: Wabayo ensonga yona yona eyakulemesa okuyamba omwana/abaana bo nga balekeire okusoma?*

6. How long do you think your child should stay in school? Why?

Probe: What is the least education level that you think your child should have before discontinuing school?

*Olowoza omwana alina kumala ibanga litya mwiisomero? Lwaaki?*

*Buuza: Olowooza omwana wo yandisomye paka kutuka wa nga akali kuvha kwiisomero?*

**Section 2: Life After Leaving School**

6. What did your child do right after leaving school?
   a. Probe: work, stayed at home, got married, informal training

*Omwanawo yakola atya ngha yakava kwiisomero?*

*a) Buuza: mulimo, yasighala waka, yafumbirwa,yagya mukutendekerwa ebindhi*

7. What kind of support—if any—did they receive from you, the community, or others after leaving school?
   a. Probe: counselling, taken care of, giving them work, informal training

*Buyambi ki, webubaayo, bwebafuna okuva yoli, y’ekyaalo, oba abandhi nga balekeire okusoma?*

*a) Buuza: kwogera nabo, kubalabirila, ,yagya mukutendekerwa ebindhi*

8. Looking back, what kind of support would have been helpful?

*Okulingha einhuma, buyambi ki obwandibeire obulunji?*

**Section 3: Emotional and Social Impact**

9. How did your child feel emotionally after leaving school?

Probe: Why do you think your child felt like that?

*Omwana wo yawulira atya ngha yakavha kwiisomero?*

*Buuza: Olowooza omwana wo lwaki yawulira atyo?*

10. How did you feel emotionally after your child left school?

*Wawulira otya ngha omwana wo aviile kwiisomero?*

11. Did you notice any changes in their behavior or mood afterward?
    a. Probe: isolation, substance use, sadness, confidence

*Wabonawo enkyukakyuka mu mbeera dhaibwe oba obuntu eluvhainuma?*

*a) Buuza: y’esosola, yakozesa ebilagalalagala, ennaku, obuvhumu.*

**Section 4: Community Reactions**

12. How did people in your community respond to your child leaving school?

*Abantu mu kitundhu kyo bebisa batya eri omwana wo ngha aviile kwiisomero?*

13. What were your expectations from your child after they left school?

- Probe: Girls versus boys

*Wali osubira ki mu mwana wo nga alekeire okusoma?*

*Buuza: abawaala na abalenzi*

14. Do you think boys and girls are treated differently after leaving school in your community?

*Olowooza abawala n’abalenzi babisibya bundhi ngha baviile kwiisomero mukitundhu kyo?*

15. Have you ever felt judged or criticized by others because your child/ward left school? Can you share an example?

*Wawuliraku oti bakusalire omusango oba bakuneniiza kubanga omwana wo avhiile kwiisomero? Osobola okuwa eky’okuboneraku?*

**Section 5: Reflections**

13. What kind of program would have helped your child after leaving school?
    a. Probe: vocational training, emotional support, mentorship

*Ntekateka yangeri kyi eyandhiyambye omwana wo ngha aviile kwiisomero?*

*a) Buuza: kutendekebwa bwa mumikono, buyambi bwa ngeri gyebawuliramu, oba kwogera nabo*

14. What ideas do you have to support other youth in your community who leave school early?

*Olinha bilowoozo ki ebisobola okuyamba abavhubuka abandhi mukitundhu kyo abaavha kwiisomero amangu?*

**Semi-structured In-depth Interview Guide for People who have Discontinued School**

**Topic:** Retrospective interviews to investigate the reasons for leaving school, community attitudes, stigma experiences and psychological impacts. Specifically, personal narratives will be collected to explore sensitive mental health issues.

***Omulamwa:*** *Ebibuuzo eby’okunonereza ku nsonga edha okuvha kwiisomero amangu, endowooza y’ekyaalo, okweinhamiza okubaawo ne engeri yekikyusa embera y’obuntu. Okusinga, abazaire oba abalabirila abavhubuka abaava kwiisomero amangu babuziibwa okugha ebisingaawo kumulamwa guno.*

**Introduction of the Researcher**

Hello. I am…..from Empowerment to Heal Uganda, based at Mpunde Health Center. Our team is conducting a study in Buyende to understand what life looks like for a child after they have discontinued school, and the- existing support systems which will help us to shape our interventions for support. The interview will take approximately 45 minutes. Please feel free to respond to the questions openly; there are no right or wrong answers. All your responses will be recorded. I will record the interview to ensure that I capture all details accurately, and it will be transcribed later.

*Mbalamwiisa. Ninze...............................okhuva mu Empowerment to Heal Uganda, okuvha kw’ilwaliro lye Mpunde. Ekitongole kyaiffe kili kola omusomo mu Buyende okutegera ensonga lwaki abaanha bavha kw’isomero amanghu n’obwewalikirivu obwekosa ku.*

*Ebibuuzo bida kumala akabhanga akagere ka dakkika 45. Wulira eidhembe okwilamu ebibuuzo mulwangala, tuzila kyakwilamu kituffu oba kiffu. Eby’okwilamu byaimwe byona byona bidhakugemebwa. Ndigya gema amalobozi okusobola okugeman buli kagema ku bibuuzo byaiffe obulunji, oluvhainhuma mbiize mubuwandhiike.*

District

*Disitulikiti*

County

*Isaaza*

Subounty

*Igombolola*

Parish

*Muluka*

Village

*Kyaalo*

**Background information**

Name

*Elinha*

Sex

*Ekikula*

Occupation

*Omulimo*

Marital status

*Embera y’obufumbo*

Education Level

*Okusoma*

Religion

*Eidhini*

Phone number

*Eisimu*

**Section 1: School Discontinuation**

1. What did you want to become when you were a child?
   a. Probe: farmer, teacher, shop-keeper, nurse

*Wyendha kubaaki ngha okali mwana muto?*

*a) Buuza: mulimi, musomesa, kutundha idhuuka, musawo.*

2. Do you think leaving school was your personal choice or influenced by other factors?
   a. Probe: peer pressure, family decision, other influencers

*Okulinga einhuma, olowoza okuva kwiisomero kwali kusalawo kwo, oba nsonga edindi?*

*a) Buuza: kusigulaghana, kusalawo kw’abengandha, ebindhi*

3. What were the main reasons you left school early?
   a. Probe: money issues, pregnancy, illness, family needs, academic struggles

*Nsonga kyi enkulu lwaki wava kwiisomero?*

*a) Buuza: nsonga dha sente, mabundha, bulwaire, by’etaago bya ab’engandha, ntalo dha by’akusoma.*

**Section 2: Life after Discontinuation**

4. What did your life look like right after leaving school?
   a. Probe: Did you work? Stay home?

*Obulamu bwafanana butya ngha wakava kwiisomero?*

*a) Wakola, wasighala waka?*

5. What kind of support, if any, did you receive at that time?
   a. Probe: from family, community, school, NGOs

*Buyambi bwa nkula kyi, webubaayo, bwewafuna mukaseera ako?*

*a) Okuva ab’engandha, kwiisomero, mubitongole*

6. What kind of support do you wish you had received?
   a. Probe: help getting work, someone to talk to, vocational or counseling program

*Buyambi kyi bwolowooza bwewandifunye?*

*a) Buuza: kuyambibwa kufuna mulimo, muntu wakwogera naye, kutendekebwa bya mumukono oba kubulilirwa.*

**Section 3: Emotional and Psychological Impacts**

7. Do you think boys and girls are treated the same after leaving school early?

*Olowooza bawala n’abalenzi babisibwa nekilala ngha baviile kwiisomero amandhu?*

8. Have you experienced sadness, regret, stress, or other emotional challenges related to discontinuing school?

*Wafunaku okunakuwala, okwewalikirila, oba obuzibu obundhi bwonabwona ngha bwekuusa ku kuvha kwiisomero?*

9. How do you feel about it now?
   a. Probe: Do you still think about it? Do you still regret it?

*Owulira otya buti?l*

*a) Buuza: Okakilowoozaku?, Okakyeidusa?*

10. How did you cope up with these feelings?

Probe: Have you ever used substances (alcohol, drugs) to cope with these feelings?

*Wakola otya okunalilira n’embeera edho?*

*Buuza: Wakozesaku ebilagalalagala (omwenge, endhaye) okusobola okulwanisa embera eyo?*

11. Do you feel there is any support available and accessible to you to help cope up with such feelings?

*Owulira nga obuyambi bw’okunalilira embera edho buliwo ela butukibwaaku?*

**Section 4: Reflections**

12. If a program had existed when you discontinued school, what kind of help would have been most useful to you?

Probe: vocational courses, mentorship,mental health support, skills training

*Singa wabayo entekateka eyabawo ngha wakava kwiisomero, buyambi bwa nkula kyi obwandibeire obw’omugaso gyoli?*

*Buuza: mirimo gya mumutwe, kwogera nabo, kutendekebwa gyamumikono*

13. If you were given an opportunity to change something in the education system or how the community supports students, what would it be?

*Singa owebwa omukisa okukyusa mu muwatwa ogw’okusoma mu Uganda, oba engeri ekyaalo gye kiyambamu abasomi, wandhi kyusisa ki?*

**Semi-structured In-depth Interview Guide for Teachers**

**Topic:** Retrospective interviews to investigate the reasons for leaving school, community attitudes, stigma experiences and psychological impacts. Specifically, parents or caregivers of youth who left school early are asked to provide deeper insights into the topic.

***Omulamwa:*** *Ebibuuzo eby’okunonereza ku nsonga edha okuvha kwiisomero/okulekera okusoma amangu, endowooza y’ekyaalo, okweinhamiza okubaawo ne engeri yekikyusa embera y’obuntu. Okusinga, abazaire oba abalabirila abavhubuka abaava kwiisomero amangu babuziibwa okugha ebisingaawo kumulamwa guno.*

**Introduction of the Researcher**

Hello. I am…..from Empowerment to Heal Uganda, based at Mpunde Health Center. Our team is conducting a study in Buyende to understand the reasons for leaving school early and the stigma attached to it. The interview will take approximately 45 minutes. Please feel free to respond to the questions openly; there are no right or wrong answers. All your responses will be recorded. I will record the interview to ensure that I capture all details accurately, and it will be transcribed later.

*Mbalamwiisa. Ninze...............................okhuva mu Empowerment to Heal Uganda, okuvha kw’ilwaliro lye Mpunde. Ekitongole kyaiffe kili kola omusomo mu Buyende okutegera ensonga lwaki abaanha bavha kw’isomero amanghu n’obwewalikirivu obwekosa kw’ekyo.*

*Ebibuuzo bida kumala akabhanga akagere ka dakiika 45. Wulira eidhembe okwilamu ebibuuzo mulwangala, tuzila kyakwilamu kituffu oba kiffu. Eby’okwilamu byaimwe byona byona bidhakugemebwa. Ndigya gema amalobozi okusobola okugema buli kagema ku bibuuzo byaiffe obulunji, oluvhainhuma mbiize mubuwandhiike.*

District

*Disitulikiti*

County

*Isaaza*

Subounty

*Igombolola*

Parish

*Muluka*

Village

*Kyaalo*

**Background information**

Name

*Elinha*

Sex

*Ekikula*

Occupation

*Omulimo*

Marital status

*Embera y’obufumbo*

Education Level

*Okusoma*

Religion

*Eidhini*

Phone number

*Eisimu*

**Section 1: Understanding School Discontinuation**

1. What do you think are the most common reasons why students discontinue school in your community?
   a. Probe: economic hardship, pregnancy, caregiving, illness, academic struggles, bullying

*Olowooza nsonga ki edhabulidho lwaki abaanha abasomu bavha kwiisomero mu kitundhu kyo?*

*a) Buuza: bukalubirivu bya nfuna, mabundha, buyambi, bulwaire, ntalo dha kusoma, kutulugunizibwa?*

2. Are there particular grades or ages when students are most likely to discontinue school? Why?

*Waliwo emyaka oba ebibiina ebimaniikwa abaanha webatera okuvha/okuleka okusoma? Lwaki?*

3. Are boys or girls more likely to discontinue school? What are the key differences in their experiences?

*Kubawala, n’abalenzi bani ababonekeire okuvha/okuleka okusoma? Ndawulo kyi eliwo mu byebabitamu?*

4. What school-related factors contribute to discontinuation?
   a. Probe: teacher behavior, school environment, curriculum challenges, lack of support

*Nsonga ki edh’ekwasaganya ne’isomero edhi letera abaanha okuva/okulekera okusoma?*

*a) Buuza: mpisa ya basomesa, embera y’kwiisomero, nkyukyuka mu byebasoma, kubula buyambi*

5. When you identify a student at risk of discontinuing school, how do you usually respond?

*Wotegeera omwana ayolekeire okulekera okusoma, otela kukigemaganya otya?*

**Section 2: Support Systems and Resources**

6. Are there any school-based programs or supports in place for students at risk of discontinuing school?

*Waliyo entekateka edh’okwiisomero edhiliyo okuyamba abaanha ababonekera okuvha kwiisomero?*

7. What kind of support, if any, do students typically receive from families or the community when struggling to stay in school?

*Buyambi bwa nkula ki, webubaayo, abaanha bwebafuna okuvha eri ab’engandha, oba ekyalo ngha balikulwanisa okusighala mwiisomero?*

8. Have you taught students who returned after discontinuing school? How were they received by peers and staff? How does their age when they return affect their treatment?

*Wasomesaku abaanha abali balekera okusoma? Bayanirizibwa batya bana banaibwe n’abasomesa? Emyaka gyaibwe ngha baize gyikosa gyitya engeri gyebababisaamu?*

9. Do you think your school and your community have enough resources (teachers, materials, facilities) to keep students enrolled?

*Olowooza eisomero lyo, n’ekitundhu kyo birina eby’etaagisa ebimala( abasomesa, eby’okozesa, ebizimbe) okuyamba abaanha okwiila mwiisomero?*

**Section 3: Psychosocial and Community Impacts**

10. Have you observed changes in students’ behavior, mood, or performance before they discontinue school?

*Wabonawoku enkyukakyuka muneyiisa y’omwaanha, mbera d’obuntu, oba enkola ye ngha akaali kulekera kusoma?*

11. Do you think students who have discontinued school face stigma or negative judgment from the school or community?

*Olowooza abannha abalekera okusoma baganana n’okweinamiza, oba okusalirwa omusango okuva kwiisomero, oba mu kyaalo?*

**Section 4: Ideas for Intervention**

12. What kinds of programs do you think would help prevent school discontinuation?
    a. Probe: peer mentoring, teacher training, school meals, academic counseling, savings programs

*Olowooza ntekateka kyi edhiziyiza okulekera okusoma?*

*a) Buuza: kwebuliragana, kutendeka basomesa, okulya kwiisomero, kubulirwa kubya kusoma, kutereka sente*

13. What types of support would help those who have already discontinued school?
    a. Probe: vocational training, re-enrollment pathways, mental health services

*Buyambi bwa kika kyi obusobola okuyamba abalekera okusoma?*

*a) Buuza: kutendekebwa byamumikono, kwiilayo kwiisomero, buyambi bwa bulamu bw’obulwaire bw’akawanga*

14. If we developed a community-based program combining mental health support and livelihood training, what features would make it successful?

*Singa tukola entekateka ngha esinzila mu kyaalo ngha egaitta eby’obulamu bw’akawanga n’okubawo, bintu kyi ebisobola ogyifuula ey’omulembe?*

15. What role do you think teachers and schools should play in supporting students who have discontinued school or are at risk?
    *Mulimo kyi gw’olowooza abasomesa n’amasomero gwe basobola okola okuyamba abaanha abaavha kwiisomero oba ababonekeire okuvha yo?*
